# Nanoporous silica gel can compete with the flower stigma in germinating and attracting pollen tubes

**DOI:** 10.3389/fpls.2022.927725

**Published:** 2022-07-27

**Authors:** Giuseppe Chichiriccò, Anna Poma, Loretta Pace

**Affiliations:** Department of Life, Health and Environmental Sciences, University of L'Aquila, L'Aquila, Italy

**Keywords:** *Crocus vernus* subsp. *vernus*, *Narcissus poeticus*, Silica gel and Vycor, pollen germination, tube growth

## Abstract

To find nanoporous substrates with hydrodynamic properties useful for pollen hydration and germination, we used the glassy Silica gel and Vycor scales and pollen with different morphological and physiological traits, that of *Crocus vernus*, and that of *Narcissus poeticus*. For *in vitro* tests, the scales were spread on microscope slides, hand pollinated, and incubated. Pollen germination was evaluated with the stereomicroscope and the tube growth was explored with scanning electron microscopy (SEM). The *in vivo* tests were carried out by sprinkling the stigmas of the *Crocus* plants with Silica gel scales and immediately after having pollinated them by hand, the plants were incubated. Three hours later, the stigmas were removed and treated for observation with SEM. *In vitro* the pollen of both species germinated on Silica gel with percentages similar to those of the *in vivo* and *in vitro* controls, accumulating fibrillary material at the interface. The tubes grew perpendicular to the surface of the scales, trying to penetrate the scales to the point of flattening with the apex. On *Crocus* stigmas sprinkled with Silica gel scales, pollen developed tubes that grew to the scales rather than penetrating the papillae. The results underline the close interaction of pollen with nanoporous artificial material, so much so that its pollen tubes are attracted to the Silica scales more than to the stigma papillae that arises from a mechanism of natural selection.

## Introduction

Sexual reproduction in angiosperms (Tandon et al., 2020) occurs through some main steps, the landing and adhesion of the pollen on the receptive stigma of the flower, the growth of the pollen tube with penetration of the stigma, and growth inside the pistil to conduct the sperm cells up to the eggs for fertilization. Pollen is enveloped by the exine wall in which plants with entomophilous pollination are covered by a layer of pollenkitt including lipids, carotenoids, flavonoids, proteins, and sugars (Pacini and Hesse, 2005) that make up the interfacial layer for interactions with biotic and abiotic environment, and further interactions are controlled by other organic compounds on the exine and the underlying wall (intine) (Hiscock and Allen, 2008). The stigma, depending on its coating, is divided into three types (Heslop-Harrison, 1977; Heslop-Harrison and Shivanna, 1977; Hiscock et al., 2002), namely, wet type if coated with a watery secretion of nutrients, dry type if coated with a dry protein film, and semi-dry if it has intermediate features. A waxy layer covers the stigma and the stigmatic papillae when these are present. Pollen before and after its exposure is subject to dehydration and to preserve viability adopting biochemical and cytological strategies (Hoekstra, 1985, 1986; Hoekstra et al., 1989; Franchi et al., 2011; Pacini and Dolferus, 2019). Based on the hydration levels and the desiccation tolerance, two categories of pollen can be distinguished (Franchi et al., 2011), the orthodox that dehydrates before the anther opens and does not suffer from the post-shedding desiccation, and the recalcitrant that retains most of the water until the anther opens and suffers from the post-shedding drying which makes it short-lived, except for a few exceptions like *Crocus* pollen (Chichiriccò, 2000a,b, 2005). The adhesion of pollen on the stigma and its appropriate hydration for germination depend on morphological, structural, and biochemical features of both partners (Edlund et al., 2004; Hiscock and Allen, 2008).

*In vitro*, pollen can germinate in culture media containing adequate concentrations of sucrose and inorganic compounds that vary by species, but generally, the germinative potential is not fully expressed as it occurs *in vivo*. A comprehensive review of the various germination media used in different phyletic groups, wild and cultivated, was recently reported (Tushabe and Rosbakh, 2021). In addition to the use of culture media, the germination capacity of pollen is sometimes roughly expressed by viability tests such as the fluorochromatic reaction (Heslop-Harrison and Heslop-Harrison, 1970). Of course, the most accurate method is to pollinate the stigma and count the tubes under the microscope after staining them. With this in mind and considering the importance of pollen viability and germination for plant fertilization, an *in vitro* method to assess this potential over time would be very useful, especially for food plants (Gómez-Mena et al., 2022).

There are artificial materials consisting of microporous (0–2 nm), mesoporous (2–50 nm), or macroporous (>50 nm) structures (Polarz and Smarsly, 2002) with particular chemical–physical properties to be used in the technological field as adsorbers, catalysts, sensors, exchangers of ions, scavengers, antibacterial substrates, molecular separators, and gas traps, also finding application in the biosciences and biomedicines (Losic et al., 2006; Almáši et al., 2016; Wang et al., 2017; Ameen et al., 2019; Li et al., 2019). Silica glasses have been extensively studied (Gin et al., 2021) in relation to their use to isolate radioactive waste, as well as for their bioactivity in particular for their property of stimulating bone regeneration. In the vegetable field, these materials find application in agriculture to fertilize the soil by releasing nutrients in small doses for plant cultivation (Gin et al., 2021). These nanoporous glassy structures have a high affinity with water and, upon their contact, the water dissociates triggering various reactions and the formation of bonds such as siloxane and hydrogen bonds, and the result may be to form a gel layer (Gin et al., 2021). It is interesting to remember that pollen shares some applications with artificial nanoporous materials due to the properties of the exine sporopollenin. This is chemically unassailable and has a variously microporous or cross-linked structure (Halbritter et al., 2018) which, after the chemical removal of the other pollen components, provides a microcapsule for applications in biomedicine and biotechnology (Mackenzie et al., 2015; Iravani and Varma, 2021), and can also be used for ion exchange chromatography (Mackenzie et al., 2015). For their property of trapping water and making it available in small doses, the Silica glass materials could act as interactive substrates for plant systems that suffer from the rehydration necessary for germination and growth, such as the pollen. In this regard, we verified this potential by using nanoporous glassy materials, namely, Silica gel and Vycor. The first (Ignaczak et al., 2003) is a granular material consisting of silicon and oxygen atoms which alternate forming a network of small cavities, and it is widely used for ion exchange chromatography, water filtration, removal of metal ions from aqueous solutions, and, of course, as a moisture absorber. The second (Gelb and Gubbins, 2000; Nordberg, 2006) consisting of B_2_O_3_ (3%), Na_2_O (0.40%), and SiO_2_ (96%) finds numerous applications including the filtration and separation of compounds, and its ability to absorb quickly organic contaminants is used as a non-contaminating getter. For germination tests, we used the pollen from two monocotyledonous plants, well-known for their luxurious flowers, *Crocus vernus* Hill subsp. *vernus* and *Narcissus poeticus* L., for the following reasons. Pollen of *Crocus* (Chichiriccò, 2000a, 2004) requires controlled hydration to preserve viability and unlike *Narcissus* pollen (Chen and Ueda, 1977), it is very difficult to germinate *in vitro*; on the stigma, its germination is affected by ambient humidity. In both species, the pollen is rich in the pollenkitt coating which was previously analyzed in C. *vernus* subsp. *vernus* (Chichiriccò et al., 2019), and it is recalcitrant in the *Crocus* and orthodox in the *Narcissus* (Chen and Ueda, 1977; Chichiriccò, 2000a), although the stigma is dry in both species and covered with a waxy layer cuticle (Heslop-Harrison and Shivanna, 1977).

## Materials and methods

### Plant material

*C. vernus* Hill subsp. *vernus* plants from the Gran Sasso (AQ, Italy) and *N. poeticus* L. plants from the Rocche highlands (AQ, Italy) were transplanted in pots during the anthesis. The stigmatic papillae of *Crocus* are receptive to pollen already a few days before the opening of the flower; however, we carried out the pollinations when the flowers disclosed, and the pollen used for each test and the respective control was removed from the same anther.

### *In vitro* germination tests

About hundred granules of Silica gel scales with 60 A° pore diameter (Baker J.T.) and Corning Vycor, 7930 Porous Glass scales with 40 A° pore diameter were scattered separately on slides under a stereomicroscope. By using a beard hair glued to the apex of a pen, pollen grains 1–2 at a time were transferred from *Crocus* anthers on the scales until an average number of 67 pollen grains was reached for each test which was repeated nine times with Silica gel and five times with Vycor. The slides were incubated in humidified Petri dishes for 3 h at 20°C and then the germinated pollen grains were evaluated with the stereomicroscope Leica Wild M10 (refer to Supplementary file 1). This incubation method, as previously tested (Chichiriccò et al., 2019), is optimal for both *Crocus* and *Narcissus* pollen, including hand-pollinated pistils. Pollen was considered germinated when its tube was at least as long as the diameter of the pollen. As for *Narcissus* pollen, the pollination method was the same but the tests were carried out only on Silica gel scales using a lower number of pollen grains. This was due to the difficult handling of the pollen that is much smaller than *Crocus* pollen, and by contact, it is easily electrified, splashing off more than *Crocus* pollen. The average number of pollen grains deposited on the scales with the beard hair was 47 for each test which was repeated three times.

For observations at scanning electron microscopy (SEM), small slides were covered on the ventral side with double-sided tape and glued with the dorsal side to the adhesive stubs, and then Silica gel or Vycor scales were spread on the ventral side of the slide. The scales were pollinated, incubated in humidified Petri dishes for 3 h at 20°C, and then dehydrated and coated with gold, as described earlier.

### *In vivo* control tests

The control tests for *Crocus* pollen were *in vivo* performed, as previously described (Chichiriccò and Picozzi, 2007; Chichiriccò et al., 2019). In short, *Crocus* plants were stripped of their tepals and anthers and wrapped with aluminum foil including the bulb and leaving the stigmas uncovered. Each plant was glued horizontally on a microscope slide (2.5 × 7.5 cm) with double-sided tape (refer to Supplementary file 2), taking care to arrange the stigmatic branches for pollination. Pollination was performed under a stereomicroscope, by depositing with the above beard hair 1–2 pollen grains on each stigma papilla until all observable papillae were pollinated. An average number of 42 pollen grains was placed on the stigmas of each plant; in total, the pollinated plants were nine. After pollination, the slides were incubated for 3 h at 20°C in moistened Petri dishes and then some drops of aniline blue and a cover slide were placed on the stigmatic branches to evaluate under a Zeiss fluorescence microscope equipped with filter set 09 (Kho and Bear, 1968), the germinated pollen grains detectable by the fluorescent pollen tube. As for *Narcissus*, due to the size and structure of the pollen and stigma, the control tests were not possible *in vivo* not even removing the pistil. Therefore, a liquid medium consisting of 5% sucrose, 0.01% H_3_BO_3_, 0.01% KNO_3_, 0.01% Ca Cl_2_ ∙ 2H_2_O, and pH 5.8 (Chichiriccò et al., 2019) was used by sowing on its pollen grains and incubating for 3 h at 20°C. Then, droplets of the incubated medium were immediately transferred to slides for the counting of the germinated pollen under the microscope light, and the total number of examined pollen grains was 220.

### Stigmas *in vivo* pollinated after spreading silica gel scales on its papillae

*Crocus* plants were prepared and placed on the 2 × 7.5 cm microscope slides as described for the *in vivo* controls, with a difference concerning the location of the stigmatic branches, and these were arranged with double-sided tape on a 2 × 1.5 cm slide which was previously placed on the end side of the microscope slide (refer to Supplementary file 2). These microscope slides bearing the plants were placed under a stereomicroscope, and Silica gel scales were placed between the stigma papillae. Soon after, pollen grains were placed with the beard hair between the scales and the papillae. The pollinated plants were incubated into moistened Petri dishes for 3 h at 20°C, after which the stigma branches were carefully cut at their base and the small slide bearing these branches was placed on the adhesive stub for dehydration, gold coating, and observation with SEM, as described earlier. Silica gel scales with the germinated pollen grains remained attached to the stigma branches during the procedures for observing with SEM.

### Scanning electron microscopy (SEM)

For SEM observations, two electron microscopes were used, namely, Philips XL30/CP and SEM-FEG Gemini 500 (Zeiss). In the first case, the samples obtained as described below were fixed in acetic acid and alcohol (1:3), dehydrated in an ethanol series until 100%, and critical point dried in CO_2_. They were then gold-coated using Balzer's SCD 040 sputtering and observed. In the second, the samples were fixed as above, dehydrated in alcohol series until 100% and in hexamethyldisilazane (HMDS), and coated with sputtering Cr target (Q 150T ES).

### Statistical analysis

Differences in the proportion of germinated pollen were tested using a binomial ANOVA approach. Standard ANOVAs referring to Gaussian distributions are not appropriate to compare proportions, as they follow binomial distributions. With the binomial ANOVA approach, a logistic model is initially built using a generalized linear model (GLM) with a binomial distribution. Then, the output of this model is used to perform an ANOVA, in which *P*-values are established using deviances and chi-squared distributions tests. We implemented this approach with the functions GLM and ANOVA in R version 3.5.2. Tukey *post-hoc* tests were done using the R package multcomp (refer to Supplementary file 3).

## Results

### *In vitro* tests

The pollen from *Crocus* and *Narcissus* when it was deposited on the Silica gel and the Vycor scales adhered to them and in the following minutes, it developed a tube. The germination percentages are reported in Table 1.

**Table 1:** Percentages (averages ± SD) of germinated pollen on Silica gel and Vycor scales, and in controls.

**% of germinated pollen**				**Comparisons**		
	**Silica**	**Vycor**	**Control**	**Silica vs. control**	**Vycor vs. Silica**	**Vycor vs. control**
*C. vernus*	83.0 ± 4.6 (9)	50.4 ± 12.0 (5)	85.9 ± 3.6 (9)	*z* = −1.131, *P* = 0.493	*z* = −9.929, *P* <0.001	*z* = −9.486, *P* <0.001
	603	338	379			
*N. poeticus*	81.0 ± 1.3 (3)	n.t	80.9 ± 1.0 (3)	*z* = −0.014, *P* = 0.989	–	–
	141		220			

*Number of replicates are given in parenthesis and below the number of pollen grains used. Comparisons refer to Tukey post-hoc tests*.

**Figure 1:**
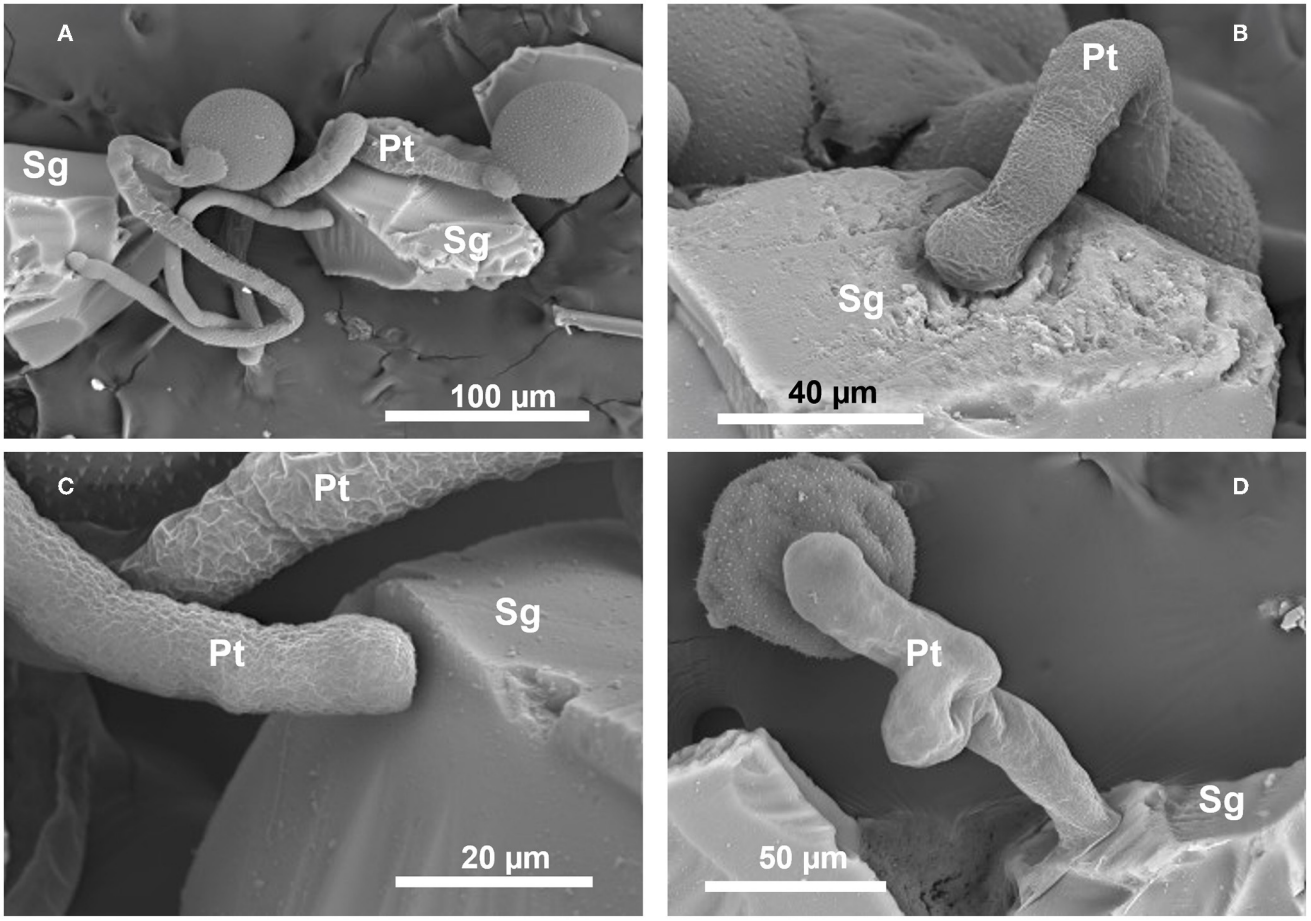
Scanning electron micrographs of *in vitro* germination and tube growth (Pt) of *Crocus vernus* subsp. *vernus* pollen on the Silica gel scales (Sg) at SEM. **(A)** Long pollen tubes. **(B)** Pollen tube growing with the apex inside a hollow of the Silica scale. **(C)** Pollen tube with flattened apex. **(D)** Extensive flattening of the tube.

**Figure 2:**
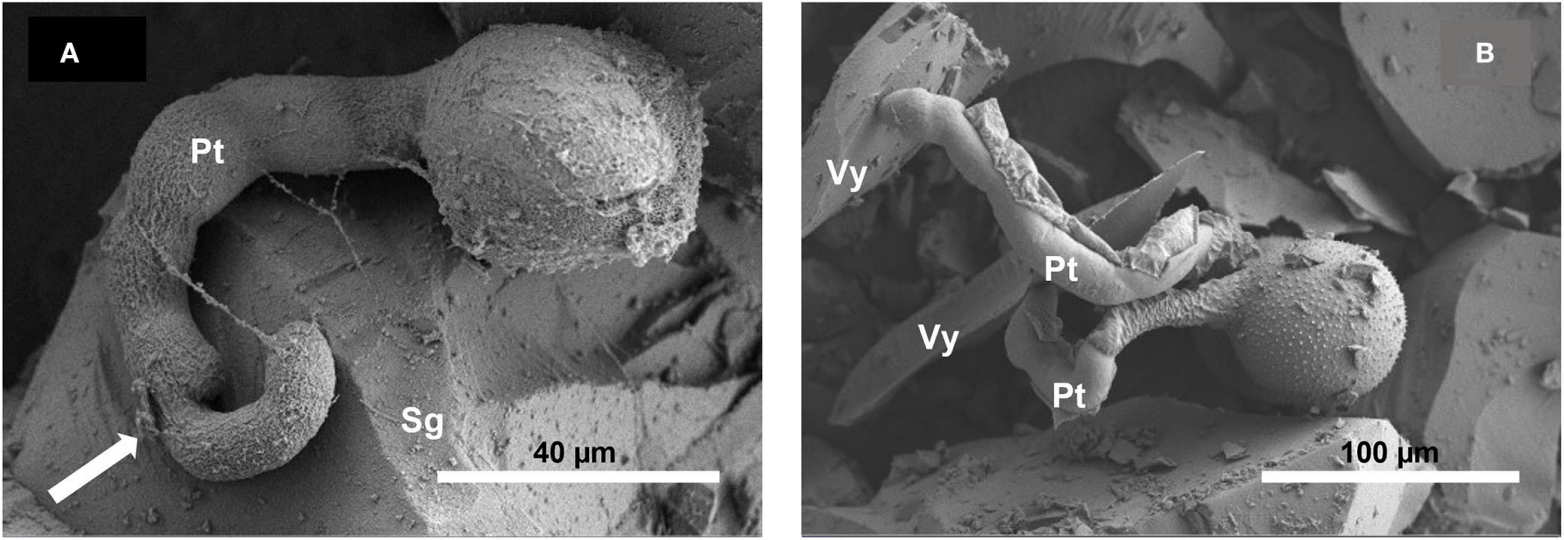
Scanning electron micrographs of *in vitro* germination of *Narcissus* pollen on Silica gel scales **(A)** and of *Crocus* pollen on Vycor scales **(B)** at SEM. In **(A)** the ramification of the tube (arrow) and in **(B)** the fracture and unwinding of the outer layer of the tube.

**Figure 3:**
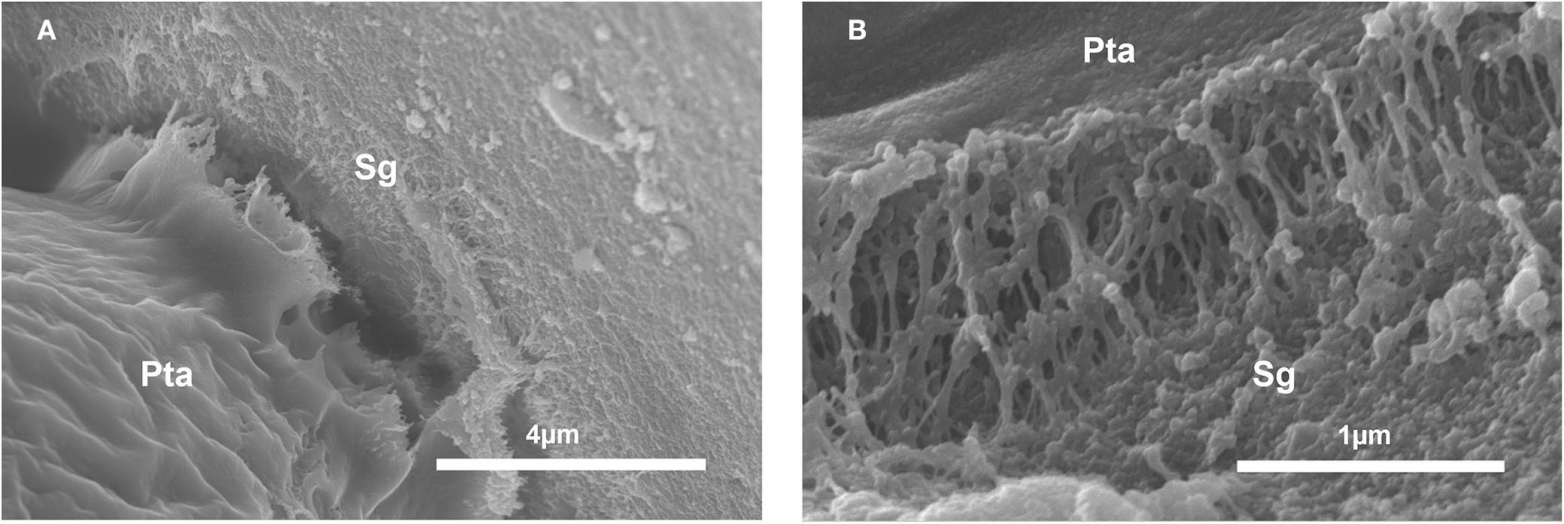
Scanning electron micrographs of *in vitro* germination of *Crocus* pollen on Silica gel scales (Sg). **(A)** Note the protrusion from the tube apex (Pta) of a material resembling a secretion. **(B)** Interface tube-Silica scale showing a mucillaginous fibrillary material forming adhesive bridges between tube apex (Pta) and Silica scale.

For *Crocus*, the *post-hoc* tests indicated that the proportion of germinated pollen in Silica (83.0%) did not differ from that observed in the control (85.9%; *z* = −1.131, *P* = 0.493), whereas the proportion of germination observed in Vycor (50.4%) was significantly lower than those observed in Silica (*z* = −9.929, *P* < 0.001) and control (*z* = −9.486, *P* < 0.001). For *N. poeticus*, the proportion of germinated pollen in Silica (81.0%) did not differ from that of control (80.9%) (*z* = −0.014, *P* = 0.989) (Table 1). Pollen developed tubes (Figure 1) which elongated up to over 600 μm, and it often developed a bifurcate tube or two pollen tubes (Figure 2). The tube apex (Figure 1) grew perpendicular to the surface of the scales by pressing and squeezing against it, and flattened sometimes extending beyond the tube apex (Figure 1D). The outer layer of the tube wall tended to fracture and separate from the tube itself which appeared as if it had been discarded (Figure 2B). A mucilaginous fibrillar secretion accumulated in the interfaces between the apex of the pollen tube and the Silica gel (Figure 3) and between the pollen tube and the Vycor (data not shown), forming numerous bridges that made the tube tightly joined to the scales. This mucilaginous secretion was observed on Silica gel and Vycor only at the point of contact with the pollen tube and nowhere else.

### *In vivo* control tests of pollen germination

*Crocus* pollen germinated within 20 min and entered the stigma papilla with the tube observable with the fluorescence microscope (refer to Supplemental file 4). The average percentage of pollen grains with the tube was 85.9% (Table 1).

### Pollen behavior on the papillae sprinkled with silica gel scales

**Figure 4:**
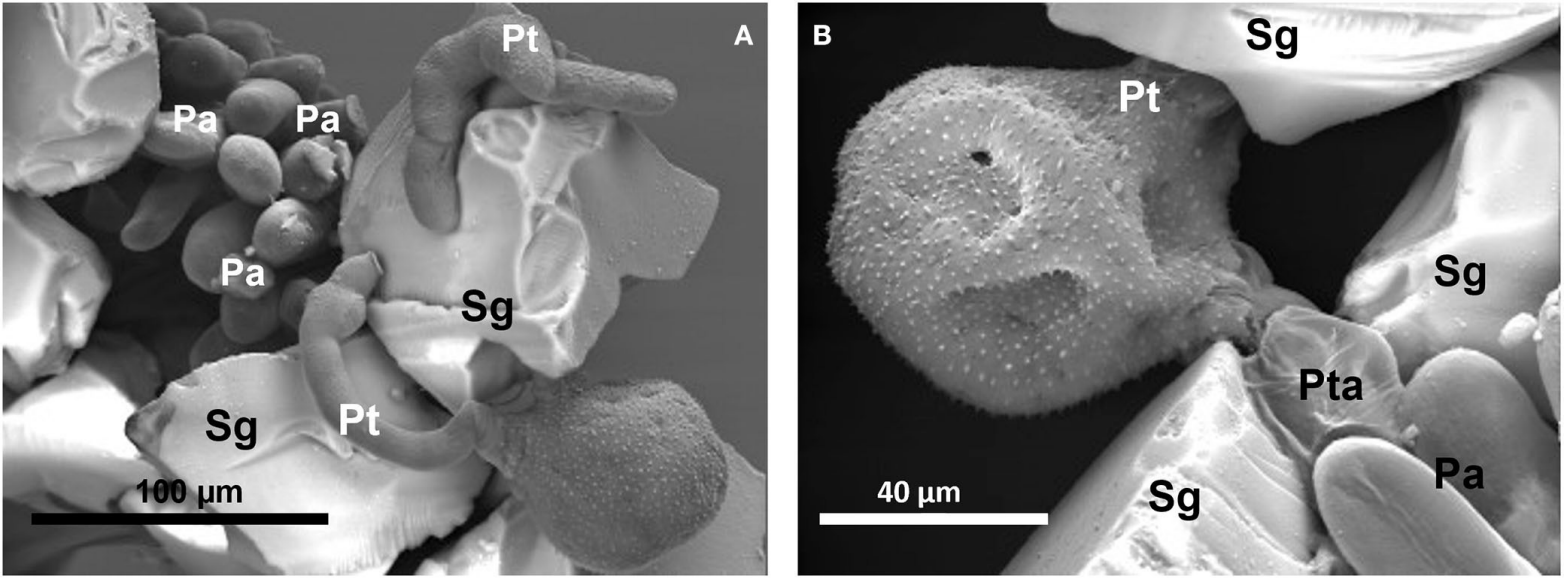
Scanning electron micrographs of the germination of *Crocus* pollen on the stigma papillae (Pa) sprinkled with Silica gel scales (Sg) before pollination. **(A)** Pollen grain with two tubes (Pt) embracing a Silica scale close to the papillae. **(B)** Pollen grain with two tubes, the emerging one is on a Silica scale and the other is closest to the papillae but its apex is flattened on the scale.

When the Silica gel scales were distributed between the papillae and then the stigmas were pollinated, pollen grains successfully germinated, and most of our observations with SEM showed tubes with the apex crushed on the scales (Figure 4), and only a minority showed tubes with the apex penetrated into the papillae. Considering that the number of effective observations with SEM was not statistically significant, they were not subjected to statistical analysis.

## Discussion

The results underline that both the recalcitrant pollen from *Crocus* and the orthodox pollen from *Narcissus* are able to interface with nanoporous Silica gel, with high performances in terms of adhesion, germination, and tube growth on the Silica gel. The first deduction from an applicative point of view is the possibility of using them as systems to test the germinative potential of pollen, which is very useful to know for crossbreeding programs. From a more strictly biological point of view, the growth dynamics of the tube that extends erect on the scales trying to penetrate them with the apex as if it were on the stigma of the flower is surprising. The tube apex due to the thrust of the growing wall and its elasticity tends to crush and flatten on the scales. Morphological, chemical, and hydrodynamic characteristics of the Silica scales can be evoked for this pollen behavior. Silica gel is known to form many hydrogen bonds in contact with water (Rimsza and Du, 2018; Gin et al., 2021) and its silanolic groups could form hydrogen bond-like bridges with pollen and its pollen tube, as well as weaker bonds such as Van der Waals forces which are recurrent in the attraction of pollen from the stigma (Ito and Gorb, 2019a). A role could be played by the changes that water undergoes, particularly at the interface, when confined within the small pores of the Silica gel and Vycor (Fouzri et al., 2002; Gin et al., 2021). The pollenkitt coating abundant in both species (Chichiriccò et al., 2019) could provide a suitable means for adhering pollen to Silica gel; according to many studies (Lin et al., 2015; Prisle et al., 2019), it plays a key role in the pollen hydration and germination. However, as previously reported (Chichiriccò et al., 2019), the pollenkitt of both *Crocus* and *Narcissus* is not essential for the hydration and germination of the pollen as this, after pollenkitt removal, is able to germinate on the papilla with percentages not significantly different from those of pollen with pollenkitt. Ito and Gorb (2019b) suggested that the success of *in vivo* pollen performance depends on the capillarity between the coalescing stigma papillae with the formation of capillary bridges which rise the stigmatic secretion toward the pollen. In addition to the papillae, capillary bridges have been reported following the adhesion of pollen to other substrates (Ito and Gorb, 2019b). Such a capillary force could be exerted by the Silica gel pores supplying water in compatible doses for the pollen and with a high chemical potential since the Silica gel absorbs solute-poor vaporized water from the environment. According to some studies (Rimsza and Du, 2018), the diffusion of water into the Silica gel is slow and controlled by the Silica ability to continuously reform a network of hydrogen bonds with water (structured water). These hydrodynamics could play a role in activating metabolically the pollen that is programmed to germinate on dry stigma where it can hydrate slowly. Furthermore, they may be related to the detachment of the external wall of the pollen tube which, by absorbing water with a high water potential, is exposed to turgor fluctuations during growth (Zonia and Munnik, 2011). However, the hydrodynamics of the porous structure alone does not explain the high pollen performance; some details are derived from Vycor's low ability to support pollen germination despite its glassy structure with Silica gel-like pores. Hence, other factors associated with the particular movement of water in the porous structure are responsible for the maximum expression of the germinative potential of pollen. *In vitro*, inorganic factors such as calcium, magnesium, potassium, and especially boron are successfully used to stimulate pollen germination (Tushabe and Rosbakh, 2021). In the present case, there are no other compounds other than silicon dioxide to which a stimulating action can be attributed. Extensive studies need to shed light on these hypotheses which are currently only speculative.

The interaction of the pollen tube of *Crocus* with the Silica gel, as well as with Vycor, manifests at the interface between them, with an accumulation of material that allows the tube tip to remain firmly attached to the scales. This study does not reveal the origin of this interfacial medium, and it could be a secretion of the tube or a modification of the tube apex or both. Anyhow, the observation that the hypothesized secretion occurs only at the point of contact of Silica gel and Vycor with the pollen and nowhere else excludes that it is due to the basic interaction between the Silica materials and the absorbed water. It is of probable pectic nature, resembling the network of the disaggregate pectic middle intine wall of *Cupressus arizonica* pollen that we observed at SEM (Chichiriccò and Pacini, 2008) following prolonged hydration. No data on this type of interaction are available in the literature.

The pollen in addition to germinating successfully as it does on the stigma grows with a prolonged autotrophic phase during which the tubes can exceed the length of 600 μm and these can either fork or even form in pairs from the same pollen grain. The bifurcation of the tube is known in spermatophytes but is not frequent in angiosperms and occurs during the growth of the tube inside the pistil (Adhikari et al., 2020); the development of two tubes, in contrast, is less known in the literature. We found the bifurcation of the pollen tube and the pollen developing two pollen tubes some time ago in the sterile triploid *Crocus sativus* L. (saffron) both *in vitro* (Chichiriccò and Grilli Caiola, 1982) and *in vivo* after intraspecific and interspecific pollinations (Chichiriccò and Grilli Caiola, 1984). Recently, we also found the bifurcation of the tube in the pollen of *C. vernus* subsp. *vernus* germinated on the papillae (refer to Supplemental file 4). In this regard, the structure of the intine plays a role in being uniformly thick in the pollen of *Crocus* (Chichiriccò, 1999; Furness and Rudall, 1999) and, therefore, there is no specific point for the development of the tube, but to develop two tubes, one should hypothesize an extranumerary division of the pollen cells to form two vegetative cells, and possibly two generative cells, it would be interesting to test this hypothesis in a future study. All these unpredictable abilities of the pollen observed in this study, also considering the low reserves of starch and sucrose in *Crocus* pollen (Chichiriccò, 2000a), highlight the role that nanoporous structures can play in stimulating cell growth. Surprisingly, the attraction of pollen by the Silica gel, as well as the development of two tubes, also manifests when the pollen is placed on the stigma partially scattered with the Silica scales, making Silica gel antagonist of the papillae in attracting tubes to themselves. We recently hypothesized that in *Crocus* (Chichiriccò et al., 2019), the release of secretion from the cuticle of the papilla (Heslop-Harrison, 1977) could occur through a network of channels similar to that proposed for foliar cuticle whose pore radius varies from a few to more than 20 nm (Schreiber, 2005; Kerstiens, 2006; Eichert and Goldbach, 2008). If the hypothesis is correct, one could deduce that the pores of the Silica gel resemble those of the papilla cuticle; in this view, the availability of a structure ready to adequately hydrate the pollen could be a competitive factor.

## Conclusion

A relevant point that emerges from this study is the high ability of the Silica gel to trigger pollen germination and attract pollen tubes, and even more, relevant the interaction with the Silica scales persists when they are placed on the stigma becoming competitors of the stigma papillae which are structurally and physiologically derived from a long path of natural selection. The high pollen yield is also manifested by the exceptional development of two pollen tubes. To the best of our knowledge, this study is the first to report a similar interaction of artificial nanoporous material with pollen to emphasize its potential in the botanical field to extend to other apical growth systems such as fungal hyphae, in addition to plant roots. If our observations will be extensible to pollen from other species, a new method of testing the germinative potential of pollen can be planned with Silica gel.

## Data availability statement

The original contributions presented in the study are included in the article/Supplementary material, further inquiries can be directed to the corresponding author/s.

## Author contributions

GC conceived and designed the experiments. GC and LP performed the experiments. AP analyzed the data. LP contributed in the images processing. All authors contributed to the article and approved the submitted version.

## Funding

This work was funded by University of L'Aquila, Department of Life, Health and Environmental Sciences 2020–2022.

## Conflict of interest

The authors declare that the research was conducted in the absence of any commercial or financial relationships that could be construed as a potential conflict of interest.

## Publisher's note

All claims expressed in this article are solely those of the authors and do not necessarily represent those of their affiliated organizations, or those of the publisher, the editors and the reviewers. Any product that may be evaluated in this article, or claim that may be made by its manufacturer, is not guaranteed or endorsed by the publisher.
