# Supplementary material for: Nanoporous silica gel can compete with the flower stigma in germinating and attracting pollen tubes
Source: Front Plant Sci. 2022 Jul 27;13:927725. doi: 10.3389/fpls.2022.927725 (PMC9363783; doi:10.3389/fpls.2022.927725)
Supplement: Supplementary file 1 [file Data_Sheet_1.PDF]

Supplementary file 1

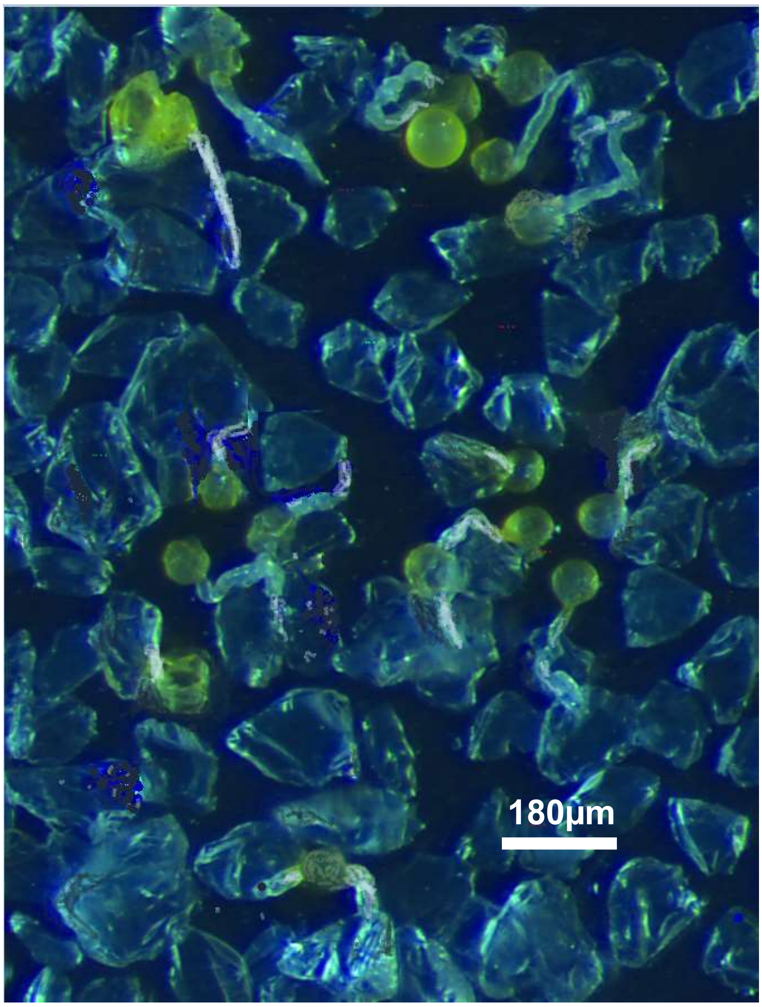

Observation with the stereomicroscope of *Crocus* pollen with tubes growing on the Silica gel scales
