# Supplementary material for: Nanoporous silica gel can compete with the flower stigma in germinating and attracting pollen tubes
Source: Front Plant Sci. 2022 Jul 27;13:927725. doi: 10.3389/fpls.2022.927725 (PMC9363783; doi:10.3389/fpls.2022.927725)
Supplement: Supplementary file 2 [file Data_Sheet_2.PDF]

Supplementary file 2

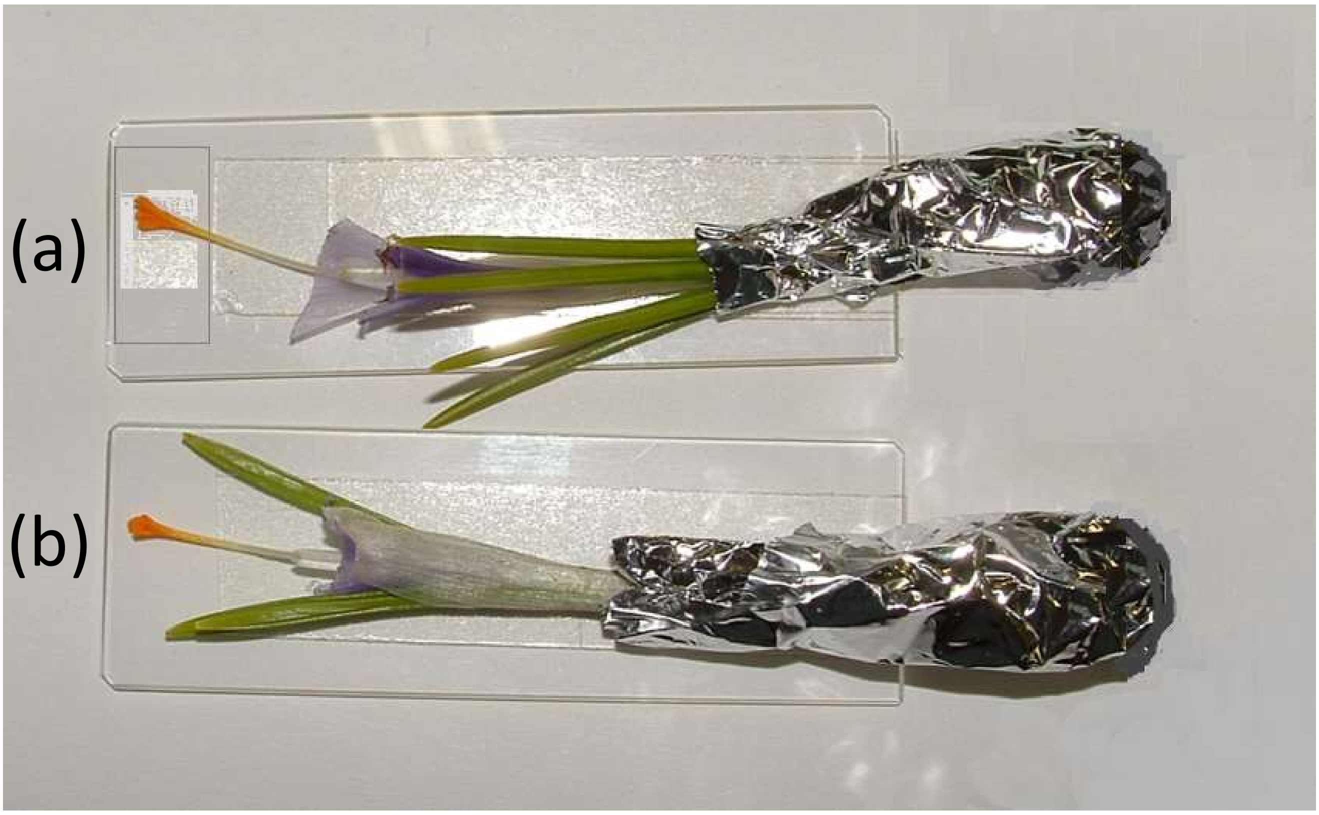

Crocus plants stuck on microscope slides (75 mm by 25 mm).

a, the microscope slide with a small slide that is removable together with the stigma attached to it, for observation with SEM.

b the slide for *in vivo* tests of the percentage of pollen germination with florescence microscope
