# Supplementary material for: Nanoporous silica gel can compete with the flower stigma in germinating and attracting pollen tubes
Source: Front Plant Sci. 2022 Jul 27;13:927725. doi: 10.3389/fpls.2022.927725 (PMC9363783; doi:10.3389/fpls.2022.927725)
Supplement: Supplementary file 3 [file Data_Sheet_3.PDF]

Supplementary file 3

Results of GLMs with binomial distribution for proportion of germinated pollen in *Crocus vernus* and *Narcissus poeticus* under different treatments. In both cases, control was used as reference. For *Crocus*: null deviance: 160.751 on 22 degrees of freedom, residual deviance: 26.316 on 20 degrees of freedom. For *Narcissus*: null deviance: 0.194 on 5 degrees of freedom, residual deviance: 0.194 on 4 degrees of freedom.

	Estimate	Standard error	Z-value	P-value
Crocus vernus				
**Intercept**	1.773	0.146	12.160	<0.0001
**Silica**	-0.205	0.181	-1.131	0.258
**Vycor**	-1.726	0.182	-9.486	<0.0001
Narcissus poeticus				
**Intercept**	1.444	0.172	8.418	<0.0001
**Silica**	-0.004	0.274	-0.014	0.989

Results of ANOVAs performed on the GLM results

	Df	Deviance	Residual degrees of freedom	Residual Deviance	P-value (Chi-square)
Crocus vernus					
**Null**			22	160.751	
**Factor**	2	134.440	20	26.316	<0.0001
Narcissus poeticus					
**Null**			5	0.194	
**Factor**	1	0.0002	4	0.194	0.989

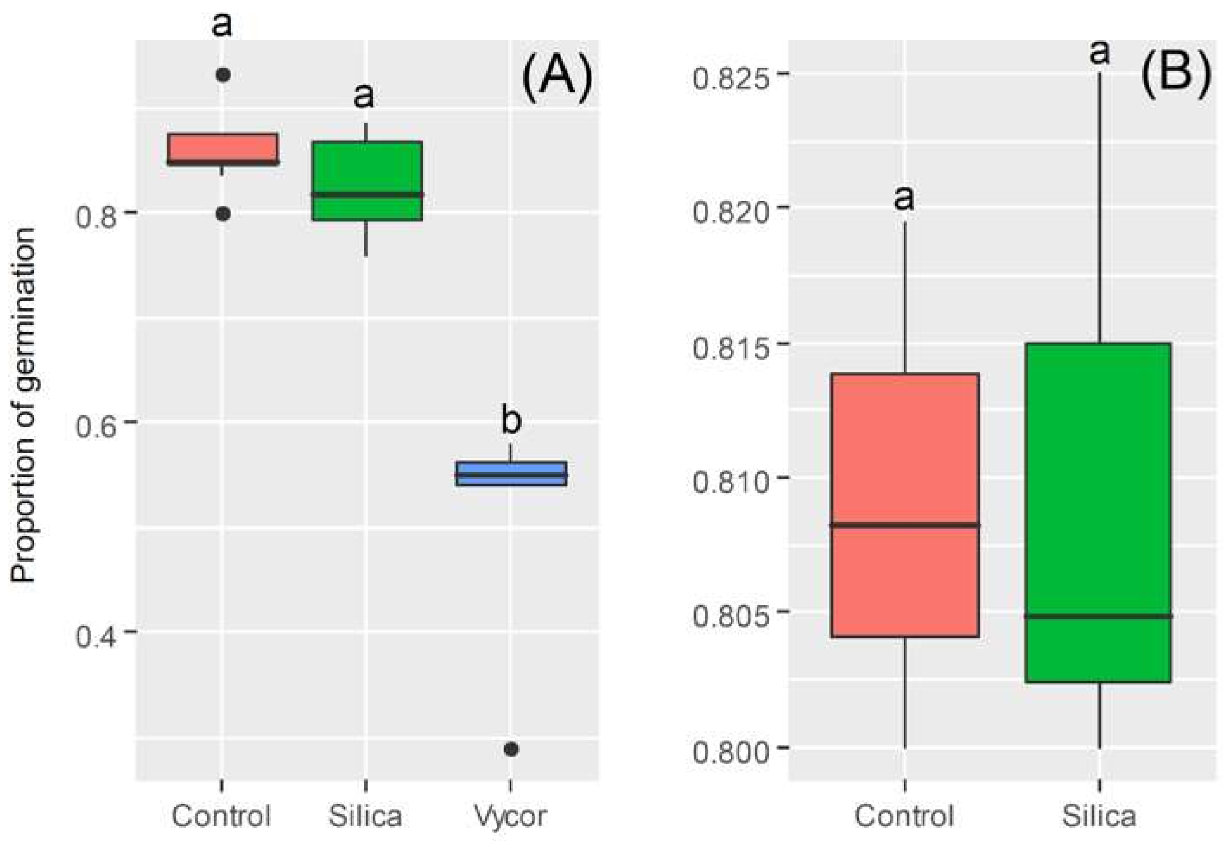

Box plots (median, quartiles, ranges and outliers) comparing proportion of germinated pollen of *Crocus vernus* (A) and *Narcissus poeticus* (B) under different treatments. Letters indicate significant differences in Tukey post-hoc tests at the $P < 0.05$ level, following ANOVAs.
