# Supplementary material for: Nanoporous silica gel can compete with the flower stigma in germinating and attracting pollen tubes
Source: Front Plant Sci. 2022 Jul 27;13:927725. doi: 10.3389/fpls.2022.927725 (PMC9363783; doi:10.3389/fpls.2022.927725)
Supplement: Supplementary file 4 [file Data_Sheet_4.PDF]

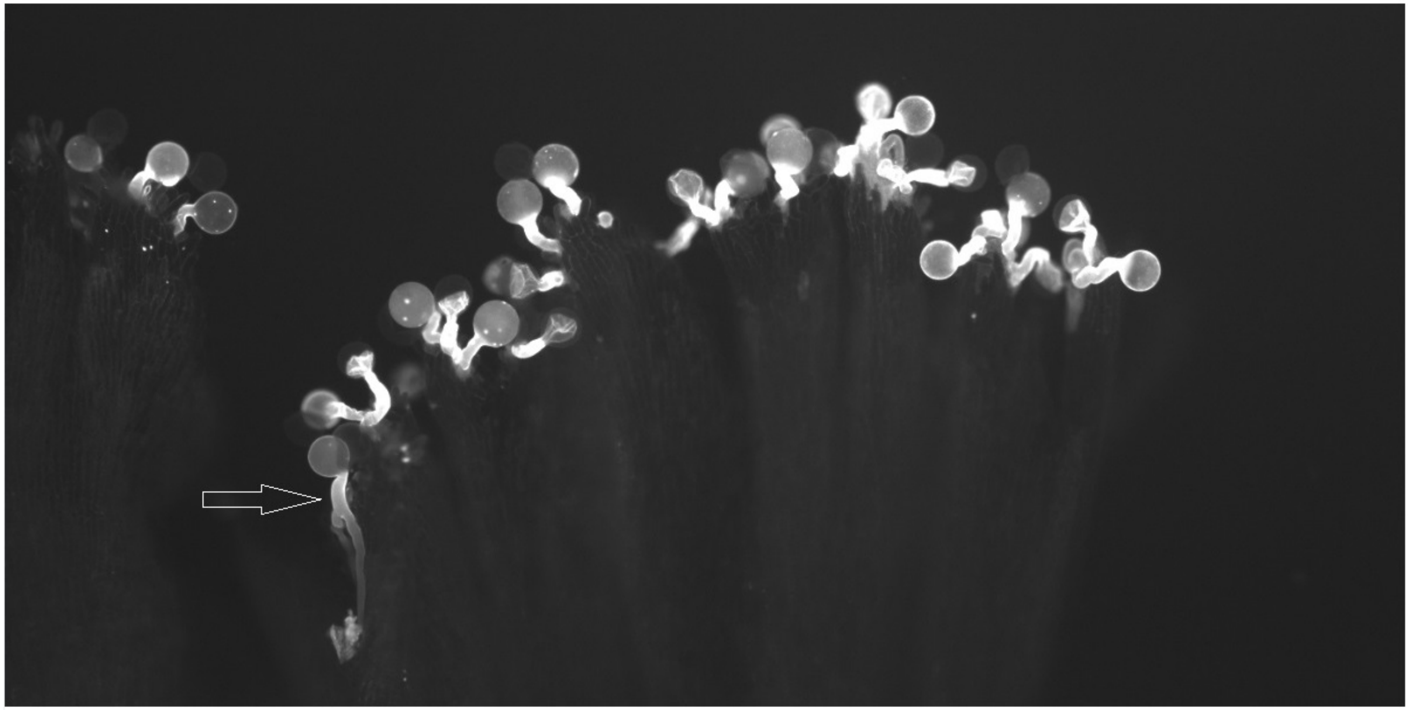

Pollen of *C. vernus* ssp *vernus* germinated on the stigma papille after hand pollination, staining with aniline blu and observed with the fluorescence microscope. Note among the fluorescent pollen tubes, a bifurcated tube (arrow).
